# Nutritional evaluation of *Zanthoxylum bungeanum* leaves and their effects on reproductive performance, milk composition, antioxidant status, and gut microbiota in sows

**DOI:** 10.5713/ab.250892

**Published:** 2026-04-28

**Authors:** Hanshu Lin, Shanchuan Cao, Heng Yin, Jianfei Zhao, Jingbo Liu

**Affiliations:** 1 College of Life Sciences and Agri-forestry, Southwest University of Science and Technology, Mianyang, China

**Keywords:** Antioxidant, Gut Microflora, Reproductive Performance, Sow, *Zanthoxylum Bungeanum* Leaves

## Abstract

**Objective:**

Two experiments were conducted to investigate the digestible energy of *Zanthoxylum bungeanum* leaves (ZBL) and effects on sow reproductive performance.

**Methods:**

In Exp 1, 24 sows (parity 3) on d 60 of gestation were used in a 10-dfecal collection study to determine the digestible energy of ZBL. Exp 1 comprised two dietary treatments: a basal diet and a 10% ZBL diet. In Exp 2, 60 sows (parity 3) on d 30 of gestation were used to evaluate reproductive performance and related indices. Exp 2 comprised two dietary treatments: a basal diet (CON group) and a 4% ZBL diet (ZBL group). The lactation period was twenty-eight days. Microbial samples were collected on farrowing day.

**Results:**

The digestible energy of ZBL was 13.77 MJ/kg. Dietary supplementation with 4% ZBL had no effect on litter size, number of live-born piglets, number of healthy piglets, litter weight at birth, or individual birth weight in sows. Milk fat concentration was higher in the ZBL group than in the CON group (p<0.05). Serum blood urea nitrogen was elevated on lactation day in the ZBL group (p<0.05), and glucose concentration was higher on weaning day in the ZBL group than in the CON group (p<0.05). Total antioxidant capacity increased on lactation day (p<0.05) and weaning day (p<0.05), while malondialdehyde decreased (p<0.05) on lactation day in the ZBL group than in the CON group. At the genus level, 4% ZBL elevated (p<0.05) the relative abundances of *Escherichia-Shigella*, *Cellulosilyticum, norank_f_Erysipelotrichaceae*, *Fecalibacterium*, *Adlercreutzia* and *Akkermansia*, and reduced (p<0.05) those of *Lachnospiraceae_XPB1014_group*, *Rikenellaceae_RC9_gut_group*, *Prevotellaceae_UCG-001* and *Bacteroides*.

**Conclusion:**

The digestible energy of ZBL for sows was 13.77 MJ/kg. Dietary supplementation with 4% ZBL partially enhanced antioxidant status and modulated fecal microbiota, but had no effect on the sow reproductive performance.

## INTRODUCTION

Pork constitutes a critical source of human protein and energy, dominating global meat consumption. Within modern swine operations, sows’ management is pivotal, as improved reproductive performance directly increases piglet output and advances the entire supply chain. During gestation and lactation, sows experience intense oxidative stress from high metabolic demands [1], which reduces feed intake, diminishes milk yield, and ultimately compromises reproductive performance [2]. Dietary antioxidant attenuates oxidative stress and improves sow reproductive performance [3]. Consequently, dietary supplementation with effective antioxidants is warranted to prevent excessive oxidative stress during gestation and lactation. Numerous studies have demonstrated that flavonoids exhibit pronounced efficacy in alleviating oxidative stress [4]. *Zanthoxylum bungeanum* leaves (ZBL) are rich in flavonoids [4].

*Zanthoxylum bungeanum* is a deciduous small-arboreal species [5] widely used in culinary sectors for its pronounced pharmacological value [6]. During harvest, the infructescences were customarily collected with leaves and branches. However, only the pericarps served as the principal raw material. Consequently, large quantities of by-products—including ZBL, seeds, and bark—were generated. These residues were frequently discarded or incinerated, leading to environmental pollution and substantial resource wastage. As the main by-product, ZBL contained nutrients (calcium, phosphorus, lipids, proteins, dietary fiber, and iron). The ZBL are rich in flavonoids such as trifolin, quercetin, and rutin [4]. Flavonoids, as potent free-radical scavengers, attenuate oxidative stress [4]. Based on this bioactivity profile, ZBL exhibited potential as a dietary ingredient for sows by exerting significant antioxidant activity, thereby mitigating oxidative stress during gestation and lactation. However, little is known about the ZBL used in sow diets. Therefore, the present study evaluated the digestible energy (DE) value and practical efficacy of ZBL in sows through measurements of DE, backfat thickness, reproductive performance, composition of sow colostrum and milk, serum biochemical indices, serum immune indices, serum antioxidant indices, and gut microflora. This provided a scientific basis for its rational incorporation into sow diets.

## MATERIALS AND METHODS

### Exp 1

*Animals, diets, and environment:* A total of 24 Landrace× Yorkshire sows (parity 3) on d 60 of gestation were housed individually in stainless steel metabolism cages each equipped with a feeder and a nipple drinker. Room temperature was maintained at 20±2°C. All sows were in good health and had similar body condition at the start of the trial. Sows were randomly allocated to one of two dietary treatments using a randomized complete block design with 12 replicate sows per treatment.

Each sow received 2.4 kg of feed daily, split into two equal meals offered at 08:00 and 18:00. Sows had free access to water at all times. Following a 5-d dietary adaptation, feces were totally collected from d 6 to 10. Chromic oxide was administered on d 6 and d 11 to mark the collection start and end points, respectively, as adapted from Liu et al [7].

Two diets were formulated: a corn-soybean meal (SBM) basal diet and an experimental diet. The basal diet, based on corn and SBM, was formulated to meet the nutrient requirements of sows [8]. The experimental diet was formulated similarly to the basal diet but included 10% ZBL, which replaced an equal portion of the basal diet. The ingredient composition and nutrient profile were presented for ZBL (Table 1) and the basal diet (Table 2). The amino acid (AA) composition of ZBL was previously determined by the same research group [4].

*Sample collection:* Each sow received 300 g of diet containing 5% Cr_2_O_3_ at 08:00 on d 6 and d 10, followed by the remaining 900 g ration for the meal. The diets provided to sows on d 1 and d 5 did not contain Cr_2_O_3_. All feces excreted from the first to the second green marker excretion were collected. Personnel conducted 24-hour shift monitoring to prevent fecal loss. The collected samples were immediately frozen at −20°C. After completion of collection, the feces from each sow were pooled, dried at 75°C for 72 h, subsequently ground through a 1-mm sieve, and stored at −20°C for chemical analysis.

### Exp 2

*Animals, diets, and environment:* A total of 60 Landrace× Yorkshire sows (parity 3) on d 30 of gestation were housed individually in 60 pens each equipped with a feeder and a nipple drinker. Room temperature was maintained at 20±2°C. All sows were in good health and had similar body condition at the start of the trial. Sows were randomly allocated to one of two dietary treatments using a randomized complete block design with 30 replicate sows per treatment.

The trial commenced on d 30 of gestation and lasted until d 28 of post-farrowing when the piglets were weaned. Daily feed allowances followed the farm’s standard gestation feeding regimen: 2.4 kg/d from d 30 to d 90 and 3.0 kg/d from d 91 to d 106. On d 107, sows were moved to farrowing crates and the feed allowance was reduced stepwise: 2.5 kg/d on d 107 and 2.0 kg/d from d 108 to farrowing. Diets were prepared once daily and offered in equal halves at 08:00 and 16:00. During lactation, sows in both treatment groups were offered the same diet, which was formulated to meet the nutrient requirements of sows [8]. Lactation feed was provided *ad libitum*. By selecting only sows bred on the same day, the farrowing interval was naturally limited to within one day. All piglets were uniformly weaned at 28 days of age.

*Two diets were formulated:* A corn-SBM basal diet and an experimental diet. The basal diet was formulated to meet the nutrient requirements of sows [8]. In the preliminary trial, a reduction in feed intake was observed in some sows when the ZBL inclusion exceeded 5%. To avoid compromising sow performance through excessive dietary levels, the experimental diets were formulated to contain 4% ZBL. The experimental diet was formulated similarly to the basal diet but replaced with 4% ZBL, which was added at the expense of corn, SBM, and bran. The ingredient composition and nutrient profile of the basal diet and the experimental diet were presented (Table 3).

*Backfat thickness measurement:* The backfat thickness (P2, 6 cm from the midline at the head of the last rib) of sows was recorded by an ultrasonic device (Piglog105; SFK Technology A/S) on gestation d 30, d 60, d 90, and on the weaning day (d 28 post-farrowing).

*Reproductive performance evaluation:* The following reproductive parameters were recorded for each sow: litter size, number of live-born piglets, number of healthy piglets, number of weak piglets, litter weight at birth, individual birth weight, and farrowing duration. At trial initiation, 30 sows per dietary treatment were enrolled. However, only 48 farrowing crates were available in a single farrowing house. To eliminate potential room effects, all sows were farrowed in a single room. Reproductive data were collected from 24 sows per treatment. No sow delivered fewer than eight piglets; consequently, data from all 48 sows were included in the statistical analysis.

*Samples collection:* Fecal samples from each sow were collected by rectal stimulation on farrowing day. After collection, each sample was transferred into a sterile 2-mL cryovial and snap-frozen in liquid nitrogen that had been pre-chilled. To minimize environmental microbial contamination, the rectal area was thoroughly cleaned before sampling each sow. Within each treatment group, feces from every three sows were pooled to constitute a single composite sample. Upon completion of the entire sampling procedure, all cryovials were stored at −80°C for chemical analysis.

After farrowing, approximately 50-mL of colostrum was manually collected from the first and last productive glands on both sides of the sow (24 sows per treatment). Samples were snap-frozen at −20°C for subsequent analyses. Neonatal piglets were allowed to suckle ad libitum from birth for energy acquisition and thermoregulation. On lactation d 21, oxytoxin 20 IU oxytocin was administered via the ear vein and approximately 30-mL of mature milk was hand-expressed (24 sows per treatment). Milk samples were immediately stored at −20°C for chemical analysis.

On farrowing day and weaning day, blood samples (5-mL) were collected from the auricular vein of each sow at 08:00. Serum was separated by centrifugation at 1,300×g for 15 min at 4°C, transferred to 1.5-mL microcentrifuge tubes, and stored at −20°C for chemical analysis.

### Chemical analysi*s*

*Nutrient analysis*: The ZBL, diets, and fecal samples were dried in a forced-air oven. Each type of sample was then ground separately through a 1-mm screen and thoroughly mixed prior to chemical analyses. The nutrient compositions of ZBL, diets, and fecal samples were analyzed according to the AOAC [9]. Dry matter (DM) was determined by oven drying at 105°C for 24 h (method 930.15; AOAC [9]). Crude protein (CP) was analyzed by the Kjeldahl method using an automatic analyzer (K1100; Hanon Advanced Technology Group) following AOAC (method 976.05; AOAC [9]). Ether extract (EE) was determined by Soxhlet extraction with petroleum ether (method 920.39; AOAC [9]). AA profiles were analyzed by high-performance liquid chromatography (Agilent-1260; Agilent Technologies) after hydrolysis with 6 mol HCl at 110°C for 24 h (method 994.12; AOAC [9]). After wet-ash sample digestion (method 975.03; AOAC [9]), the calcium content was determined by inductively-coupled plasma spectroscopy according to the method of Cao et al [10], and the phosphorus content was determined by inductively-coupled plasma spectroscopy according to the method of Zhao et al [11]. Neutral detergent fiber (NDF) and acid detergent fiber (ADF) were determined using a fiber analyzer (Ankom Technology). The gross energy (GE) of feed and fecal samples was determined using an automatic isoperibolic oxygen bomb calorimeter (Parr 1281; Automatic Energy Analyzer). Total flavonoid and polyphenol contents in ZBL were determined according to Lei et al [12]. The ZBL (1.0 g) was extracted with 22-mL of 50% ethanol under sonication for 1 h, followed by centrifugation at 7,012×g for 5 min. The residue was extracted twice more, and the three supernatants were combined and diluted to 100-mL. Total flavonoid content was determined using the sodium nitrite–aluminium nitrate colorimetric method. The extract was reacted sequentially with sodium nitrite, aluminium nitrate, and sodium hydroxide solutions, and the absorbance was measured at 510 nm. Rutin served as the standard. Total polyphenol content was determined by the Folin–Ciocalteu method. The extract was mixed with the Folin–Ciocalteu reagent and sodium carbonate solution, incubated in the dark, and measured at 778 nm. Gallic acid was used as the standard.

*Composition of sow colostrum and milk:* In Exp 2, the concentration of colostrum and milk including fat, protein, urea nitrogen and lactose was determined by a fully automated milk analyzer (Milko ScanTM FT+Analyzer; Foss). The fully automated milk analyzer was validated using standard sow milk samples of known composition to ensure measurement accuracy.

*Serum biochemistry:* Serum concentrations of glucose (GLU), total protein (TP), total cholesterol (TC), triglycerides (TG), low-density lipoprotein cholesterol (LDL), high-density lipoprotein cholesterol (HDL), blood urea nitrogen (BUN), as well as the activities of alanine aminotransferase (ALT) and aspartate aminotransferase (AST) were determined with an automatic biochemical analyzer (HITACHI-7020; Hitachi). The corresponding assay kits were supplied by Shandong Boke Biotechnology.

*Serum immunology:* The concentrations of immunoglobulin A (IgA), immunoglobulin M (IgM), and immunoglobulin G (IgG) in serum of sows were determined by ELISA kits (Nanjing Jiancheng Bioengineering Institute) following the manufacturer’s instructions.

*Serum antioxidant status:* The activities of catalase (CAT), glutathione peroxidase (GSH-Px), total superoxide dismutase (T-SOD), total antioxidant capacity (T-AOC), and the concentration of malondialdehyde (MDA) in serum of sows were determined by reagent kits (Aidisheng Biotechnology) followed the manufacturer’s instructions.

### Gut microflora analysis

Microbial profiling of the gut microbiota was performed by targeting the 16S rRNA gene and was conducted. Total microbial DNA was extracted from approximately 200 mg of cecal contents using a commercial DNA extraction kit (Qiagen). DNA quality and concentration were evaluated by agarose gel electrophoresis and NanoDrop spectrophotometry (Thermo Fisher Scientific). The V3–V4 regions of the bacterial 16S rRNA gene were amplified using primers 341F and 806R. Purified amplicons were pooled at equimolar concentrations and sequenced on an Illumina MiSeq platform (2×300 bp) at Majorbio Bio-Pharm Technology. Raw sequencing data were quality-filtered, merged, and clustered into operational taxonomic units (OTU) at 97% sequence similarity. Taxonomic classification was performed using the SILVA 16S rRNA database (release 138). The OTU table was normalized by rarefaction prior to downstream analysis. Fecal microbiota data were subjected to significance tests for between-group differences using similarity analysis (ANOSIM) and multi-response permutation procedures (MRPP); differential taxa between groups were identified by linear discriminant analysis (LDA) effect size (LEfSe) at multiple taxonomic levels.

### Calculations and statistical analysis

The apparent nutrient digestibility and DE of the test ingredient were calculated as follows:


(1) $$\begin{array}{l} \text{Apparent digestibility of energy in the test diet }(\%)=\\ ([\text{GE of the test diet}\times \text{Total feed intake}]-[\text{GE of feces}\times \\ \text{Total fecal excretion}])/(\text{GE of the test diet}\times \\ \text{Total feed intake}]\times 100 \end{array}$$

(2) $$\begin{array}{l} \text{Apparent digestible energy of the test diet }(\text{MJ}/\text{kg})=\\ \text{GE of the test diet}\times \text{Apparent digestibility of energy}\\ \text{in the test diet}]/100 \end{array}$$

(3) $$\begin{array}{l} \text{DE of the test ingredient }(\text{MJ}/\text{kg})=\\ (\text{Apparent DE of the test diet}-[1-\text{Replacement}\\ \text{proportion of test ingredient for basel diet}]\times \\ \text{Apparent DE of the basal diet})/\text{Replacement proportion}\\ \text{of test ingredient for basal diet}\text{.} \end{array}$$

All statistical analyzes were conducted using SPSS (2008) statistical software (ver.17.0 for windows; SPSS). Data on backfat thickness, reproductive performance colostrum, milk, and serum indices collected in Exp 2 were likewise analyzed by a Student’s t-test with p<0.05 considered statistically significant.

## RESULTS

### Digestible energy of *Zanthoxylum bungeanum* leaves

In Exp 1, for the control group, the feed intake was 12 kg, the fecal output was 2.01 kg, the GE content in the feces was 15.74 MJ/kg, and the DM content of the feces was 91.29%. For the group fed the diet containing 10% ZBL, the feed intake was 11.31 kg, the fecal output was 1.86 kg, the GE content in the feces was 16.14 MJ/kg, and the DM content of the feces was 91.40% (Table 4). The DE of ZBL for sows was 13.77 MJ/kg, the apparent total tract digestibility of energy was 83.14% and the apparent total tract digestibility of DM was 84.35% (Table 5).

### Backfat thickness and reproductive performance

Dietary supplementation with ZBL had no influence on the backfat thickness of sows (Table 6). Reproductive performance parameters, including litter size, number of live-born piglets, number of healthy piglets, number of weak piglets, litter weight at birth, individual birth weight, and farrowing duration were not affected by the inclusion of 4% ZBL in the diet (Table 7).

### Composition of sow colostrum and milk

Dietary supplementation of ZBL had no effect on the concentrations of fat, protein, urea nitrogen, and lactose of colostrum in sows (Table 8). However, ZBL increased the fat concentration of milk in sows (p<0.05). The ZBL had no impact on the concentrations of protein, urea nitrogen, and lactose.

### Serum biochemical indices

Dietary supplementation of ZBL elevated serum BUN concentration on lactation day (Table 9, p<0.05), whereas the concentrations of GLU, TP, TC, TG, LDL, HDL, and the activities of ALT, and AST in sow serum remained unaffected. The ZBL elevated serum GLU concentration on weaning day (p<0.05), whereas the concentrations of TP, TC, TG, LDL, HDL, BUN, and the activities of ALT, and AST in serum of sows remained unaffected.

### Serum immune and antioxidant indices

The ZBL had no effect on serum concentrations of IgA, IgG, and IgM in sows at either lactation day or weaning day (Table 10).

Dietary supplementation of ZBL increased serum T-AOC and decreased MDA concentration on lactation day (Table 11, p<0.05), whereas the activities of CAT, GSH-Px, and T-SOD were not affected. The ZBL increased serum T-AOC (p<0.05), while no effects were observed on the activities of CAT GSH-Px, T-SOD, or on the concentration of MDA.

### Gut microflora

**Figure 1:**
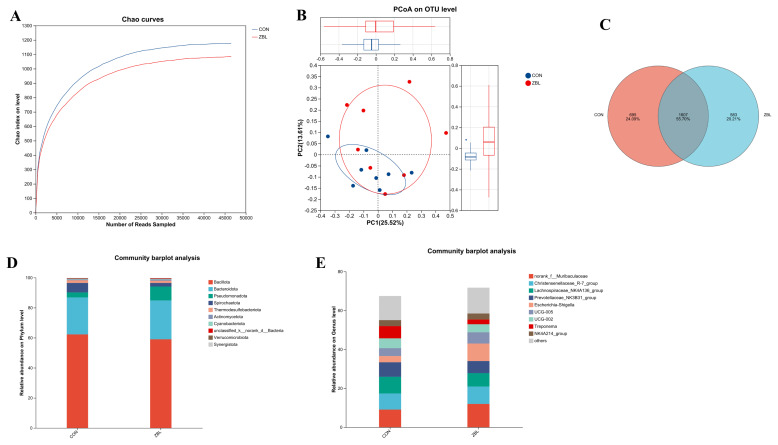
The effect of feed *Zanthoxylum bungeanum* leaves on gut microbiota diversity of sows (n = 8). (A) Chao index on levels; (B) Principal coordinates analysis (PCoA) on operational taxonomic unit (OTU) level; (C) Venn diagram; (D) Microbial community barplot analysis at the phylum level; (E) Microbial community barplot analysis at the genus level. CON = control group; ZBL = 4% *Zanthoxylum bungeanum* leaves group.

Rarefaction curves (Figure 1A) confirmed adequate sequencing depth for both CON and ZBL groups. Principal coordinates analysis (PCoA, Figure 1B, p<0.05) revealed distinct microbial community structures. Venn analysis (Figure 1C) identified 2,302 OTU in CON and 2,190 in ZBL, with 695 CON-specific, 583 ZBL-specific, and 1,607 shared OTU. These data suggested that dietary ZBL can modulate the number of microbial species in the sow gut.

At the phylum level (Figure 1D), the top five dominant phyla in sows were *Bacillota*, *Bacteroidota*, *Pseudomonadota*, *Spirochaetota*, and *Thermodesulfobacteriota*. At the genus level (Figure 1E), the five most abundant genera were *norank_f_Muribaculaceae*, *Christensenellaceae_R-7_group*, *Lachnospiraceae_NK4A136_group*, *Prevotellaceae_NK3B31_group*, and *Escherichia–Shigella*.

**Figure 2:**
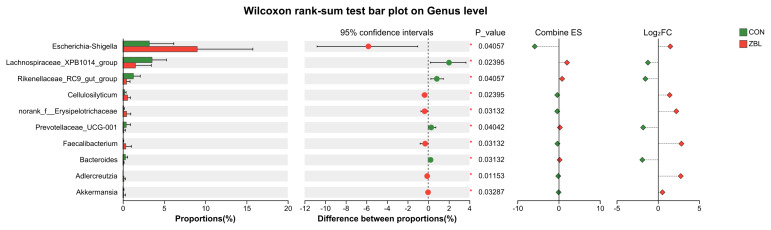
The Wilcoxon rank-sum test of differential bacterial genera in sows feed *Zanthoxylum bungeanum* leaves (n = 8). CON = control group; ZBL = 4% *Zanthoxylum bungeanum* leaves group.

Wilcoxon rank-sum tests at the genus level (Figure 2) showed that the relative abundances of *Escherichia-Shigella*, *Cellulosilyticum*, *norank_f_Erysipelotrichaceae*, *Fecalibacterium*, *Adlercreutzia*, and *Akkermansia* were higher in the ZBL group than in the CON group (p<0.05), whereas those of *Lachnospiraceae_XPB1014_group*, *Rikenellaceae_RC9_gut_group*, *Prevotellaceae_UCG-001*, and *Bacteroides* were lower (p<0.05).

**Figure 3:**
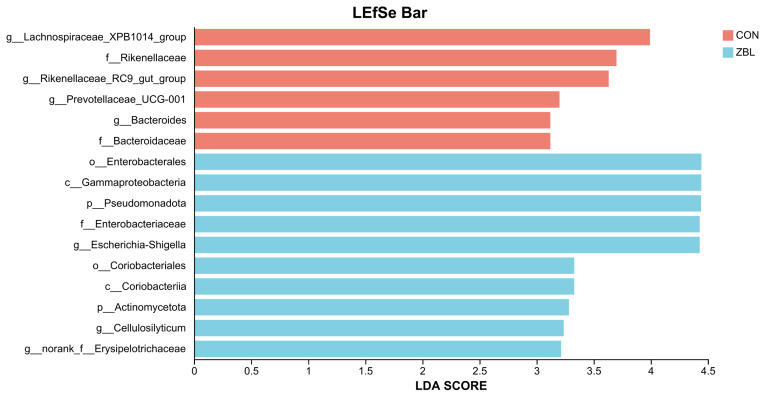
Linear discriminant analysis (LDA) effect size (LEfSe) analysis of dominant bacterial genera in sows feed *Zanthoxylum bungeanum* leaves (n = 8). CON = control group; ZBL = 4% *Zanthoxylum bungeanum* leaves group.

LEfSe (LDA scores>3.0, p<0.05, Figure 3) indicated that the CON group was enriched in *g_Lachnospiraceae_XPB1014_group*, *f_Rikenellaceae*, *g_Rikenellaceae_RC9_gut_group*, *g_Prevotellaceae_UCG-001*, *g_Bacteroides*, and *f_Bacteroidaceae*. In contrast, the ZBL group was enriched in *o_Enterobacterales*, *c_Gammaproteobacteria*, *p_Pseudomonadota*, *f_Enterobacteriaceae*, *g_Escherichia-Shigella*, *o_Coriobacteriales*, *c_Coriobacteriia*, *p_Actinomycetota*, *g_Cellulosilyticum*, and *g_norank_f_Erysipelotrichaceae*.

## DISCUSSION

The ZBL contain a broad spectrum of nutrients, including calcium, phosphorus, lipids, proteins, dietary fiber, and iron. In addition, they are rich in flavonoids such as trifolin, quercetin and rutin [4]. The ZBL used in the present experiment contained 53.31 g/kg total flavonoids and 43.37 g/kg total polyphenols. Numerous studies demonstrate that flavonoids potently alleviate oxidative stress [4]. The ZBL exhibit potential as a dietary ingredient for sows. However, there have been no relevant studies to report their incorporation into sow diets until now.

The results of this study showed that the DE of ZBL for sows was 13.77 MJ/kg. The apparent total tract digestibility of energy was 83.14%. The DE is a major component for evaluating the energy values of swine diets [13]. The DE and apparent digestibility of a feedstuff directly reflect the extent to which its nutrients are absorbed and utilized by sows. The DE is pivotal for sow performance, influencing not only productivity within a single parity but also reproductive output across subsequent parities. However, the DE system fails to account for urinary energy and heat increment, which may lead to formulation errors. The most accurate means to predict the pigs’ response to energy intake is to use diet net energy (NE) contents as model inputs and use the model to generate estimates of effective metabolizable energy (ME) for predicting the pig’s response to energy intake [8]. In the present study, the DE value of ZBL was 13.77 MJ/kg. The present study demonstrates that ZBL possess a considerable DE value for sows. Therefore, the DE values provided in this study serve as important baseline data. Future research should determine its ME through total urine collection or its NE via respiration calorimetry to fully realize its application potential.

Backfat thickness is a key indicator for assessing the body condition of sows, and its variation across gestational stages markedly influences reproductive performance [14]. Reproductive performance represents a primary economic determinant of sow productivity. In the present study, neither backfat thickness nor reproductive performance differed between treatments. Dietary inclusion of capsaicin has been reported to exert no adverse effects on the backfat thickness of gestating sows [15], a finding consistent with the results of the present study. During the trial, sows in the ZBL group exhibited faster defecation. Constipation is known to compromise sow reproductive performance, suggesting that ZBL might alleviate this condition. However, no quantitative data were collected, and the underlying mechanism warrants further investigation. As an agricultural by-product, ZBL was considerably less expensive than conventional feedstuffs. Partial substitution of the basal diet with this ingredient might therefore reduce production costs.

The quantity and quality of porcine colostrum and milk critically affect the growth performance and health status of neonatal piglets [16]. Therefore, improving sow health and immunocompetence during gestation and lactation not only enhances dam robustness and reproductive efficiency, but also strengthens the immune competence of the offspring [17,18]. In the present study, ZBL increased the fat concentration in milk. As colostrum and milk are the first nutrients supplied to neonatal piglets [19], the elevated milk fat provides additional ME. Previous reports have likewise demonstrated that phytogenic extracts can beneficially raise the energy density of sow milk [20], which corroborated our findings. Consequently, the inclusion of ZBL in the diet increased the fat concentration of sow milk, thereby enabling it to deliver more energy to piglets.

The health status and metabolic condition of an animal’s body can be reflected through serum biochemical indicators [21]. Swine serum biochemical indices are closely linked to antioxidant status [22]. The TP reflects protein nutriture, absorption and general nitrogen metabolism, whereas BUN indicates protein catabolism [23]. Transaminase plays an important role in the process of AA metabolism, and AST acts as an indicator intracellular enzyme relative to liver function and is used as the index of liver damage [24]. In the present study, dietary inclusion of 4% ZBL elevated serum BUN on lactation day, suggesting a higher rate of protein degradation. However, no changes were observed in serum TP or ALT. Collectively, while the elevated BUN suggests an influence on protein metabolism, the stability of TP and ALT indicates that dietary ZBL did not adversely affect overall metabolic homeostasis or hepatic function in sows.

Immune competence is of paramount importance for sows to resist pathogenic challenges. Quantification of serum immunoglobulins provides a reliable index of immune function [25]. The IgG constitutes approximately 75% of total immunoglobulins, while IgA is the second most abundant in serum. The IgM serves as an early indicator of infection. Collectively, these three immunoglobulins function as primary immunoglobulins in immune responses and are therefore critical to the immunological status of sows. The ZBL used in the present experiment are abundant in flavonoids. Previous studies demonstrated that dietary inclusion of 1 g/kg puerarin increased IgA and IgG levels in serum [26]. In the present experiment, inclusion of ZBL in the diets of sows exerted no significant effects on serum concentrations of IgG, IgA, or IgM. Puerarin is an isoflavone extracted from Pueraria mirifica, a wildlife leguminous plant [26]. The variability in flavonoid sources derived from different plant species may represent the primary contributor to the inconsistent experimental outcomes.

The T-AOC represents non-enzymatic antioxidant defense, and MDA reflects oxidative stress [26]. Oxidative stress disrupts endogenous protective mechanisms in sows. Flavonoids, as potent free-radical scavengers, attenuate oxidative stress [4]. The ZBL contain abundant flavonoids and possess considerable pharmacological value [4]. Dietary inclusion of 1 g/kg puerarin lowered the MDA concentration in serum of sows [26], a finding consistent with the results of the present study. In the present study, ZBL elevated serum T-AOC on lactation day and weaning day while reduced MDA concentration on lactation day. These results indicate that ZBL improved the antioxidant status of sows.

The intestinal microbiota plays a pivotal role in host health, physiology, and metabolism [27]. In sows, the maternal gut microbiome can be transmitted to the conceptus via the placenta or to neonatal piglets via colostrum and milk [28], thereby shaping offspring health and ultimately affecting sow reproductive performance. Consequently, microbial composition in the sow gut is of critical importance. In the present study, PCoA revealed distinct clustering of fecal microbial communities between treatments, and Venn analysis demonstrated that dietary supplementation of ZBL altered the number of bacterial species. This shift is presumably attributable to the potent antimicrobial activity of phenolic compounds abundant in ZBL [29], which might inhibit pathobionts and remodel the microbiota. Previous studies had identified *Bacillota* and *Bacteroidota* as the dominant phyla in the sow gut [30]. Consistently, *Bacillota* was the most abundant phylum in the current dataset, followed by *Bacteroidota*—mirroring both the sow gut consensus and the human gut profile [31]. The *Bacillota* and *Bacteroidota* ratio was positively correlated with fat mass [32] and reflected host energy metabolism. In the present trial, *Bacillota* and *Bacteroidota* constituted the most abundant phyla in the sow gut. Dietary inclusion of 4% ZBL slightly altered the gut microbiota composition. However, *Bacillota* and *Bacteroidota* remained the most abundant taxa. The *Bacillota*/*Bacteroidota* ratio has been regarded as a pivotal index of gut microbiota health. For example, a high *Bacillota*/*Bacteroidota* ratio has been repeatedly implicated in obesity and metabolic-related conditions [33]. The principal butyrate producers (e.g., *Faecalibacterium*, *Roseburia*) belong to the phylum *Bacillota*, whereas the abundance of *Bacteroidota* is linked to propionate production [34]. The relative abundance of these bacteria directly determines short-chain fatty acid (SCFA) yield. Most of the SCFA produced by gut fermentation are absorbed by the intestine and can be taken up by the mammary gland as precursors for de novo synthesis of lipids based on the net mammary uptake fluxes [35]. We hypothesized that ZBL may influence milk fat synthesis through the “microbiota-SCFA-metabolism” axis. This potential improvement in lipid utilization could represent one possible mechanism behind the concurrent significant increase in milk fat concentration observed in this study. This remains to be verified through subsequent measurements of blood SCFA concentrations and mammary metabolic gene expression. Collectively, these findings suggested that the milk fat-elevating effect of ZBL might be mediated, at least in part, through modulations of the gut microbiota and subsequent alterations in lipid metabolism, thereby potentially augmenting sow productivity. Additionally, *Bacillota* and *Bacteroidota* abundances have been reported to be inversely associated with IgA levels [36]. A modest decline in these phyla in the ZBL group might therefore account for the slight increase in serum IgA observed herein. At the genus level, the ZBL group exhibited a significant increase in the abundance of *Cellulosilyticum, Adlercreutzia, Fecalibacterium*, and *Akkermansia*. *Cellulosilyticum* is typically enriched in the gut of healthier pigs [37], indicating that ZBL ameliorated the maternal microbiota and, consequently, sow health. *Adlercreutzia* plays a key role in Glu and lipid metabolism, and its depletion is associated with metabolic dysregulation [38]. The higher abundance observed herein would therefore be expected to improve sow glycolipid metabolism, an interpretation consistent with the elevated milk fat concentration recorded in the present trial. Thus, dietary ZBL appear to enhance glycolipid utilization, thereby benefiting reproductive performance. *Fecalibacterium* and *Akkermansia* are both well-documented, anti-inflammatory commensals [39], whereas ZBL are rich in flavonoids such as trifolin, quercetin and rutin [4] that possess pronounced anti-inflammatory activity [40]. The concurrent enrichment of *Fecalibacterium* and *Akkermansia* therefore suggests that ZBL conferred an anti-inflammatory advantage to gestating and lactating sows.

## CONCLUSION

The DE of ZBL was 13.77 MJ/kg for sows. Dietary supplementation with 4% ZBL partially enhanced antioxidant status by increasing serum T-AOC and decreased MDA concentration. It also increased milk fat concentration and modulated fecal microbiota. But it had no effect on the sow reproductive performance including litter size, number of live-born piglets, number of healthy piglets, litter weight at birth, and individual birth weight. Collectively, these results indicate that while ZBL supplementation improves antioxidant status, milk composition, and gut microbiota, it does not adversely affect reproductive performance. This provides a scientific basis for its rational incorporation into sow diets.

## Figures and Tables

**Table 1:** Nutrient levels of *Zanthoxylum bungeanum* leaves (air-dry basis)

Items (%)	Content
GE (MJ/kg)	16.57
DM	91.83
CP	15.33
EE	3.58
NDF	23.48
ADF	14.36
Calcium	3.34
Phosphorus	0.14
Aspartic acid	1.25
Threonine	0.59
Serine	0.59
Glutamic acid	1.33
Glycine	0.67
Alanine	0.71
Cysteine	0.13
Valine	0.67
Methionine	0.14
Isoleucine	0.55
Leucine	1.03
Tyrosine	0.51
Phenylalanine	0.71
Lysine	0.75
Histidine	0.26
Arginine	0.70
Proline	1.07
Total 17 amino acids	11.66
Total flavone (g/kg)	53.31
Total polyphenol (g/kg)	43.37

GE, gross energy; DM, dry matter; CP, crude protein; EE, ether extract; NDF, neutral detergent fiber; ADF, acid detergent fiber.

**Table 2:** The composition and nutrient levels of the feed in Exp. 1 (air-dry basis)

Items (%)	Basal diet	Experimental diet
Corn	57.10	51.39
Soybean meal	19.50	17.55
Bran	11.80	10.62
*Zanthoxylum bungeanum* leaves	-	10.00
Fish meal	2.10	1.89
Soy oil	4.30	3.87
CaHPO_4_	1.50	1.35
NaCl	0.40	0.36
Limestone	0.80	0.72
Threonine (98%)	0.10	0.09
_L_-Lysine^•^ HCl (≥78.8%)	0.30	0.27
Choline chloride	0.10	0.09
Premix^1)^	2.00	1.80
Total	100.00	100.00
Nutrient levels (%)^2)^		
GE (MJ/kg)	15.87	15.66
DM	86.18	80.46
CP	16.12	16.04
EE	2.60	2.69
NDF	13.55	18.14
ADF	6.33	11.68
Calcium	0.86	1.11
Phosphorus	0.73	0.67

1) The premix provided the following per kg of diets: vitamin A 5,512 IU, vitamin B_6_ 3 mg, vitamin B_12_ 24 μg, vitamin D 2,250 IU, vitamin E 24 mg, vitamin K 3 mg, biotin 0.15 mg, riboflavin 6 mg, D-pantothenic acid 15 mg, nicotinic acid 20 mg, folic acid 1.2 mg, Cu (CuSO_4_·5H_2_O) 15 mg, Fe (FeSO_4_·7H_2_O) 100 mg, Mn (MnSO_4_) 30 mg, Zn (ZnO) 100 mg, Se (Na_3_SeO_3_) 0.2 mg, I [Ca(IO_3_)_2_] 0.3 mg.

2) All values were measured.

GE, gross energy; DM, dry matter; CP, crude protein; EE, ether extract; NDF, neutral detergent fiber; ADF, acid detergent fiber.

**Table 3:** The composition and nutrient levels of the feed in Exp. 2 (air-dry basis)

Items (%)	Basal diet	Experimental diet
Corn	57.10	55.70
Soybean meal	19.50	18.20
Bran	11.80	10.50
*Zanthoxylum bungeanum* leaves	-	4.00
Fish meal	2.10	2.10
Soy oil	4.30	4.30
Calcium hydrogen phosphate	1.50	1.50
Sodium chloride	0.40	0.40
Limestone	0.80	0.80
Threonine (98%)	0.10	0.10
_L_-Lysine^•^ HCl (≥78.8%)	0.30	0.30
Choline chloride	0.10	0.10
Premix^1)^	2.00	2.00
Total	100.00	100.00
Nutrient levels (%)^2)^		
DE (MJ/kg)	14.69	14.61
DM	86.18	79.33
CP	16.12	15.85
EE	2.60	2.63
NDF	13.55	17.75
ADF	6.33	11.57
Calcium	0.86	0.99
Phosphorus	0.73	0.71

1) The premix provided the following per kg of diets: vitamin A 5,512 IU, vitamin B_6_ 3 mg, vitamin B_12_ 24 μg, vitamin D 2,250 IU, vitamin E 24 mg, vitamin K 3 mg, biotin 0.15 mg, riboflavin 6 mg, D-pantothenic acid 15 mg, nicotinic acid 20 mg, folic acid 1.2 mg, Cu (CuSO_4_·5H_2_O) 15 mg, Fe (FeSO_4_·7H_2_O) 100 mg, Mn (MnSO_4_) 30 mg, Zn (ZnO) 100 mg, Se (Na_3_SeO_3_) 0.2 mg, I [Ca(IO_3_)_2_] 0.3 mg.

2) DE value was calculated, while all other values were measured.

DE, digestible energy; DM, dry matter; CP, crude protein; EE, ether extract; NDF, neutral detergent fiber; ADF, acid detergent fiber.

**Table 4:** The feed intake, fecal output, and the nutrient contents in feces of sows

Items (%)	Control	10% *Zanthoxylum bungeanum* leaves	SEM	p-value
Feed intake (kg)	12.00	11.31	0.406	0.027
Fecal output (kg)	2.01	1.66	0.238	0.191
Feces				
GE (MJ/kg)	15.74	16.14	0.277	0.004
DM	91.29	91.40	0.734	0.731

SEM, standard error of the mean; GE, gross energy; DM, dry matter.

**Table 5:** DE of *Zanthoxylum bungeanum* leaves (n = 12)

Items	*Zanthoxylum bungeanum* leaves
DE (MJ/kg)	13.77±0.62
Apparent digestibility of energy (%)	83.14±0.89
Apparent digestibility of DM (%)	84.35±0.77

Values are means±standard error.

DE, digestible energy; DM, dry matter.

**Table 6:** Effects of *Zanthoxylum bungeanum* leaves on backfat thickness of gestation sows (n = 30)

Backfat thickness (mm)	Control	4% *Zanthoxylum bungeanum* leaves	SEM	p-value
D 30 of gestation	15.47	15.74	0.156	0.227
D 60 of gestation	16.50	16.42	0.159	0.724
D 90 of gestation	16.70	16.79	0.115	0.583
Weaning day	14.49	14.71	0.156	0.316

SEM, standard error of the mean.

**Table 7:** Effects of *Zanthoxylum bungeanum* leaves on reproduction performance of sows (n = 24)

Items	Control	4% *Zanthoxylum bungeanum* leaves	SEM	p-value
Litter size (head)	13.58	13.25	0.249	0.348
Number of live-born piglets (head)	12.25	12.46	0.227	0.518
Number of healthy piglets (head)	11.58	11.75	0.200	0.553
Number of weak piglets (head)	0.67	0.71	0.154	0.849
Number of stillborn piglets (head)	1.29	0.79	0.188	0.101
Litter weight at birth (kg)	16.87	18.18	0.633	0.150
Individual birth weight (kg)	1.38	1.46	0.047	0.216
Farrowing duration (min)	262.9	267.0	6.287	0.645

SEM, standard error of the mean.

**Table 8:** Effects of *Zanthoxylum bungeanum* leaves on the composition of colostrum and milk in sows (n = 24)

Items (%)	Control	4% *Zanthoxylum bungeanum* leaves	SEM	p-value
Colostrum				
Fat	6.38	5.61	0.416	0.197
Protein	22.14	22.96	0.986	0.558
Urea nitrogen	79.33	74.00	1.915	0.055
Lactose	2.73	3.11	0.231	0.250
Milk				
Fat	5.46	6.96	0.343	0.003
Protein	5.02	5.78	0.423	0.214
Urea nitrogen	46.52	50.14	2.041	0.217
Lactose	5.72	6.23	0.463	0.443

SEM, standard error of the mean.

**Table 9:** Effects of *Zanthoxylum bungeanum* leaf on serum biochemical indices of sows (n = 24)

Items	Control	4% *Zanthoxylum bungeanum* leaves	SEM	p-value
Lactation day				
GLU (mmol/L)	3.57	3.67	0.174	0.687
TP (g/L)	63.45	64.02	2.145	0.854
TC (mmol/L)	0.85	0.94	0.115	0.588
TG (mol/L)	0.44	0.45	0.058	0.904
LDL (mmol/L)	0.56	0.45	0.060	0.222
HDL (mmol/L)	0.51	0.61	0.057	0.212
BUN (mmol/L)	2.27	2.77	0.174	0.048
ALT (U/L)	44.91	44.62	1.596	0.899
AST (U/L)	21.33	20.68	1.021	0.652
Weaning day				
GLU (mmol/L)	3.45	4.05	0.157	0.010
TP (g/L)	65.66	64.98	1.896	0.801
TC (mmol/L)	1.12	1.23	0.117	0.543
TG (mol/L)	0.49	0.48	0.053	0.907
LDL (mmol/L)	0.42	0.53	0.058	0.208
HDL (mmol/L)	0.44	0.50	0.065	0.514
BUN (mmol/L)	2.45	2.43	0.172	0.932
ALT (U/L)	44.70	48.44	1.386	0.063
AST (U/L)	21.62	19.56	1.021	0.161

SEM, standard error of the mean; GLU, glucose; TP, total protein; TC, total cholesterol; TG, triglycerides; LDL, low-density lipoprotein cholesterol; HDL, high-density lipoprotein cholesterol; BUN, blood urea nitrogen; ALT, alanine aminotransferase; AST, aspartate aminotransferase.

**Table 10:** Effects of *Zanthoxylum bungeanum* leaves on serum immune indicators of sows (n = 24)

Items (mg/mL)	Control	4% *Zanthoxylum bungeanum* leaves	SEM	p-value
Lactation day				
IgA	1.06	1.15	0.119	0.598
IgG	20.26	19.95	0.527	0.681
IgM	10.97	11.03	0.510	0.927
Weaning day				
IgA	1.19	1.25	0.115	0.714
IgG	19.75	18.96	0.333	0.101
IgM	10.15	9.86	0.331	0.542

SEM, standard error of the mean; IgA, immunoglobulin A; IgG, immunoglobulin G; IgM, immunoglobulin M.

**Table 11:** Effects of *Zanthoxylum bungeanum* leaves on serum antioxidant of sows (n = 24)

Items	Control	4% *Zanthoxylum bungeanum* leaves	SEM	p-value
Lactation day				
CAT (U/mL)	30.60	32.36	1.221	0.313
GSH-Px (U/mL)	737.3	727.8	6.491	0.306
T-SOD (U/mL)	57.42	61.70	1.964	0.130
T-AOC (nmol/mL)	2.60	2.89	0.100	0.043
MDA (nmol/mL)	2.90	2.53	0.127	0.041
Weaning day				
CAT (U/mL)	31.60	32.31	0.739	0.501
GSH-PX (U/mL)	636.8	635.0	3.797	0.734
T-SOD (U/mL)	60.83	63.46	1.266	0.148
T-AOC (nmol/mL)	2.04	2.56	0.133	0.009
MDA (nmol/mL)	3.79	3.47	0.125	0.069

SEM, standard error of the mean; CAT, catalase; GSH-Px, glutathione peroxidase; T-SOD, total superoxide dismutase; T-AOC, total antioxidant capacity; MDA, malondialdehyde.

## Data Availability

Upon reasonable request, the datasets of this study can be available from the corresponding author.
